# Genetic wealth, population health: Major histocompatibility complex variation in captive and wild ring‐tailed lemurs (*Lemur catta*)

**DOI:** 10.1002/ece3.3317

**Published:** 2017-08-17

**Authors:** Kathleen E. Grogan, Michelle L. Sauther, Frank P. Cuozzo, Christine M. Drea

**Affiliations:** ^1^ University Program in Ecology Duke University Durham NC USA; ^2^ Department of Evolutionary Anthropology Duke University Durham NC USA; ^3^ Department of Anthropology University of Colorado‐Boulder Boulder CO USA; ^4^ Lajuma Research Centre Louis Trichardt (Makhado) 0920 South Africa; ^5^ Department of Biology Duke University Durham NC USA

**Keywords:** conservation genetics, genetic diversity, immunogenetics, inbreeding, MHC‐DRB, strepsirrhine primate

## Abstract

Across species, diversity at the major histocompatibility complex (MHC) is critical to individual disease resistance and, hence, to population health; however, MHC diversity can be reduced in small, fragmented, or isolated populations. Given the need for comparative studies of functional genetic diversity, we investigated whether MHC diversity differs between populations which are open, that is experiencing gene flow, versus populations which are closed, that is isolated from other populations. Using the endangered ring‐tailed lemur (*Lemur catta*) as a model, we compared two populations under long‐term study: a relatively “open,” wild population (*n* = 180) derived from Bezà Mahafaly Special Reserve, Madagascar (2003–2013) and a “closed,” captive population (*n* = 121) derived from the Duke Lemur Center (DLC, 1980–2013) and from the Indianapolis and Cincinnati Zoos (2012). For all animals, we assessed MHC‐DRB diversity and, across populations, we compared the number of unique MHC‐DRB alleles and their distributions. Wild individuals possessed more MHC‐DRB alleles than did captive individuals, and overall, the wild population had more unique MHC‐DRB alleles that were more evenly distributed than did the captive population. Despite management efforts to maintain or increase genetic diversity in the DLC population, MHC diversity remained static from 1980 to 2010. Since 2010, however, captive‐breeding efforts resulted in the MHC diversity of offspring increasing to a level commensurate with that found in wild individuals. Therefore, loss of genetic diversity in lemurs, owing to small founder populations or reduced gene flow, can be mitigated by managed breeding efforts. Quantifying MHC diversity within individuals and between populations is the necessary first step to identifying potential improvements to captive management and conservation plans.

## 1 INTRODUCTION

In taxa as diverse as fish, amphibians, reptiles, birds, and mammals, diversity and/or the specific alleles present at the major histocompatibility complex (MHC) have become important measures of genetic health. Polymorphism at MHC loci has been linked to many proxy measures of quality (e.g., disease resistance, ornamentation, life span) or fitness (e.g., mating success, offspring production) in individuals, populations, and species (Bernatchez & Landry, 2003; Sommer, 2005). For conservation biologists, these measures reflect the potential for adaptation to emerging zoonosis, habitat fragmentation, and climate change (Ujvari & Belov, 2011). Therefore, understanding whether MHC diversity differs between wild and captive populations could be significant to the development of captive management strategies, as well as to coordinating the protection and recovery of wild populations. Here, we compare diversity at the MHC‐DRB gene between a “closed,” captive population and a more “open,” wild population. This comparison allows investigation of the influences of genetic isolation and balancing selection on MHC diversity, as well as the interplay between these forces and captive management.

Genetic diversity, defined as the number of genetic variants within a population or between individuals, allows future generations to adapt to environmental change. Loss of genetic diversity can be especially detrimental to populations that are small, fragmented, or isolated (reviewed in Frankham, 2005a, 2005b; Hedrick & Kalinowski, 2011). Such populations are generally less genetically diverse than are larger, more contiguous populations (Madsen et al., 2000; Sutton, Robertson, & Jamieson, 2015; reviewed in Ouborg, Angeloni, & Vergeer, 2010), owing to population bottlenecks, founder effects, genetic drift, or inbreeding—mechanisms that may act simultaneously and synergistically. Similarly, captive populations are often less genetically diverse than their wild counterparts. They are often founded by only a few individuals and, once established, can be considered “closed” because they experience little additional influx, either from the wild or from other captive groups (reviewed in Ivy, 2016; Fernández‐de‐Mera et al., 2009; Yao, Zhu, Wan, & Fang, 2015; but see Newhouse & Balakrishnan, 2015). Compared to captive populations, all but the most geographically isolated of wild populations are typically more “open” owing to greater gene flow. Thus, relative to wild populations, captive populations may experience more inbreeding and genetic drift and consequent loss of genetic diversity. For these reasons, captive populations can serve as important proxies for fragmented, wild populations, specifically for examining the consequences of founder effects and decades of isolation on genetic diversity. Assessments of the genetic diversity of captive populations are also critical if these populations are to serve as potential sources for reintroduction efforts for endangered species.

At the individual level, decreases in genetic diversity across the genome and at specific loci typically reduce quality or fitness (i.e., individuals that have decreased genetic diversity have poorer health, shorter life spans, and produce fewer offspring than do individuals that have greater genetic diversity: Crnokrak & Roff, 1999; Frankham, 2005a; Keller & Waller, 2002). At the population level, loss of genetic diversity reduces adaptive potential, thereby increasing the risk of extinction. The loss of genetic diversity from population decline, genetic drift, or inbreeding is thus one of the major threats to endangered species (Frankham, 2005a, 2005b). Nonhuman primates and other long‐lived species are particularly vulnerable to the negative effects of genetic loss because their slow life histories and low rate of reproduction can severely hinder the recovery of genetic diversity (Charpentier, Widdig, & Alberts, 2007). From a theoretical perspective, quantifying the existing genetic diversity within populations is essential for (1) identifying the influence of genetic diversity on quality, fitness, and adaptive potential, (2) elucidating the evolutionary pressures influencing current levels of biological variation, and (3) understanding the specific mechanisms of action. From an applied perspective, understanding the relationship between genetic diversity and fitness has become a task of increasing urgency for recovery and conservation of endangered species.

Because almost half of all nonhuman primate species are classified as “threatened” (IUCN 2010), the threat from loss of genetic diversity is of particular importance for developing successful primate conservation plans. Although transgenerational health and reproductive data are logistically difficult to obtain for long‐lived species, in a few semi‐free‐ranging primates, loss of genetic diversity has been correlated with increased health risks, including greater ectoparasite prevalence and burden, as well as decreased immunocompetence (Charpentier, Williams, & Drea, 2008; Noble, Chesser, & Ryder, 1990; Van Coillie et al., 2008). These health threats, however, did not translate into shorter life spans for less heterozygous individuals (Charpentier, Williams, & Drea, 2015). Decreasing genetic diversity is often measured using neutral heterozygosity, nH_o_, calculated from microsatellite data (Hartl & Clark, 1997; Reed & Frankham, 2001). Nonetheless, these correlations denote an indirect relationship between genetic diversity and health risks because differences in microsatellites do not change protein sequences. Therefore, nH_o_ does not equate to functional genetic diversity that reflects the adaptive potential of a population. Relative to nH_o_, genetic diversity at protein coding or regulatory regions is better estimates of evolutionary potential because they are directly associated with factors that influence individual fitness, population viability, and the ability to respond to environmental change (Hedrick, 2001; Ouborg et al., 2010; Väli, Einarsson, Waits, & Ellegren, 2008).

We could gain a better understanding of how an individual's genetic makeup affects its quality or fitness by examining genetic variation at critical functional genes, such as those of the MHC. The MHC is the most polymorphic gene family within vertebrates and is responsible for the activation of the adaptive immune system. MHC genes encode proteins that distinguish between “self” and “non‐self” peptides (Piertney & Oliver, 2006). MHC products bind and present “non‐self” peptides to immune system cells, initiating the body's immune response (Piertney & Oliver, 2006). Because each MHC molecule “recognizes” a particular subset of pathogens, both overall MHC diversity and specific MHC alleles have been linked to resistance or susceptibility to pathogens (Biedrzycka, Kloch, Buczek, & Radwan, 2011; Carrington & Bontrop, 2002; Evans & Neff, 2009; Oliver, Telfer, & Piertney, 2009; Savage & Zamudio, 2011; Wegner, Kalbe, Kurtz, Reusch, & Milinski, 2003; reviewed in Bernatchez & Landry, 2003 and Sommer, 2005). In some species, the links between MHC and disease are independent of the impact of nH_o_, providing evidence that parasite load is specifically MHC‐mediated, rather than a result of nH_o_ (Schwensow, Fietz, Dausmann, & Sommer, 2007; Westerdahl et al., 2005). Beyond the direct link between MHC and health, MHC diversity is also linked to differential lifetime survival (Huchard, Knapp, Wang, Raymond, & Cowlishaw, 2010) and reproductive success (Kalbe et al., 2009; Knapp, Ha, & Sackett, 1996; Ober, 1999; Sauermann et al., 2001; Setchell, Charpentier, Abbott, Wickings, & Knapp, 2010; Smith et al., 2010; Thoß, Ilmonen, Musolf, & Penn, 2011).

Given the correlations between MHC diversity and proxies of quality or fitness, such as health, individual and offspring survival, and reproductive success, measuring MHC diversity should be a fundamental component of wildlife conservation and captive‐breeding efforts (reviewed in Ujvari & Belov, 2011). Individuals in small or fragmented populations and populations that have undergone a bottleneck often show reduced MHC‐DRB diversity when compared to individuals in large or connected populations and in populations prior to size reduction (Agudo et al., 2011; Bollmer, Ruder, Johnson, Eimes, & Dunn, 2011; Sutton, Nakagawa, Robertson, & Jamieson, 2011; Sutton et al., 2015). A decline in genetic diversity can be further compounded by the more pronounced effects of genetic drift on smaller populations. Thus, small or fragmented populations are subject to a double‐pronged threat.

**Figure 1:**
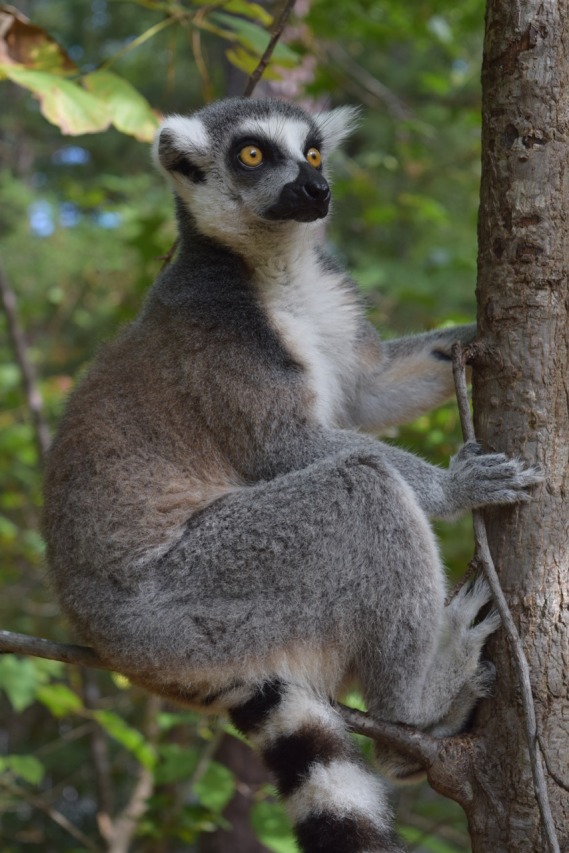
Photo of ring‐tailed lemur by CM Drea

As an endangered primate (Andriaholinirina et al., 2014), the ring‐tailed lemur (*Lemur catta*; Figure 1) is currently facing many anthropogenic stressors, including habitat fragmentation, increased contact with humans and domestic animals, and climate change (Schwitzer et al., 2013). Evaluated by the IUCN in 2014, the population density of ring‐tailed lemurs in Madagascar was found to be low and restricted to isolated fragments. Habitat loss, hunting pressure, and the illegal pet trade are estimated to have produced at least a 50% reduction in the wild population over the last few decades (Andriaholinirina et al., 2014). Population estimates from areas known to historically contain ring‐tailed lemurs indicate a dramatic population reduction to as few as 2,500 wild animals spread across southwestern Madagascar (Gould & Sauther, 2016; LaFleur et al.,^2016^), increasing the probability of loss of genetic diversity due to genetic drift. Because of these threats, ring‐tailed lemurs might be representative of vulnerable species worldwide. Moreover, the extensive, long‐term study of large populations in the wild and in captivity (Gould, Sussman, & Sauther, 2003; Jolly, 1966; Sauther, Gould, Cuozzo, & O'Mara, 2015; Sussman et al., 2012; Zehr et al., 2014), coupled with confirmed susceptibility to inbreeding depression (Charpentier et al., 2008), make the ring‐tailed lemur a suitable model for testing if functional genetic diversity at the MHC differs between captive and wild populations or if MHC diversity declines in captivity.

We used two measures of MHC diversity to compare wild and captive individuals: absolute MHC allelic diversity and the diversity of MHC supertypes, which are groups of MHC alleles with similar motifs in their binding pockets despite nucleotide differences (Doytchinova & Flower, 2005; Huchard, Weill, Cowlishaw, Raymond, & Knapp, 2008; Sepil, Lachish, Hinks, & Sheldon, 2013). These alleles therefore likely bind similar or identical pathogen peptides and can be grouped according to functional binding properties. Using these data, we evaluated the effect of breeding management on the more “closed,” captive population by estimating if its diversity (1) differs from that of the more “open,” wild population and (2) has changed across decades. We also used previously published microsatellite data to assess the relationship between nH_o_ and functional genetic diversity (or MHC‐DRB diversity: Charpentier et al., 2008; Grogan, McGinnis, Sauther, Cuozzo, & Drea, 2016; Pastorini et al., 2015), as well as to estimate effective population sizes.

## 2 METHODS

### 2.1 Subjects

Our subjects were 301 ring‐tailed lemurs, including 121 (67 females; 51 males; three infants of unknown sex) captive animals from three institutions that are part of the Association of Zoos and Aquariums (AZA) in the USA and 180 (86 females; 94 males) wild animals from Bezà Mahafaly Special Reserve (BMSR), in the region of Atsimo‐Andrefana, Madagascar.

The wild ring‐tailed lemurs at BMSR have been studied since 1987 and have been collared for identification and monitored monthly since 2003 (for more details on BMSR and the monthly census of the wild population, see Sussman et al., 2012). From 1987 to 2013, this population has generally averaged approximately 95 adult, ring‐tailed lemurs annually (range = 58–120; Gould, Sussman, & Sauther, 1999; Gould et al., 2003; Sauther & Cuozzo unpublished data). Our BMSR population represents the majority (>90%) of adults collared from 2003 to 2013. We consider this population to be “open,” owing to the known immigration and emigration of animals (Sauther, Sussman, & Gould, 1999; Sussman, 1991, 1992).

The captive ring‐tailed lemurs comprised 52 individuals residing, from 2012 to 2013, at the Duke Lemur Center (DLC; *n* = 32) in Durham, NC, the Indianapolis Zoo (*n* = 18), in Indianapolis, IN, and the Cincinnati Zoo (*n* = 2), in Cincinnati, OH, as well as an additional 69 individuals that had previously resided at the DLC, from 1980 to 2012, for which DNA samples could be obtained. These captive animals included individuals born at any of the three institutions, as well as individuals that had transferred between these and other institutions, either to address a housing constraint or to satisfy a breeding recommendation from the AZA Species Survival Plan (SSP) that originated in 1995. All individuals were housed in mixed‐sex groups, with similar housing and provisioning conditions across the three institutions (for details, see Scordato, Dubay, & Drea, 2007; McKenney, Rodrigo, & Yoder, 2015). In past decades, groups at the DLC ranged from male–female pairs to multimale, multifemale groups as large as 25 individuals. More recently, groups have included a maximum of 12 individuals. We treated the captive animals as a single population, regardless of home institution, because they are managed as such by the AZA; however, we have surveyed only three of over 200 institutions that house ring‐tailed lemurs. We also consider them a relatively “closed” population because of the small group of founders and because, each year, only a few individuals breed or transfer between institutions.

### 2.2 Sampling

We obtained whole blood or tissue collected from wild and captive lemurs by veterinarians following established protocols (Charpentier et al., 2008; Sauther & Cuozzo, 2008). All of our sampling methods followed approved animal handling guidelines and protocols of the Institutional Animal Care and Use Committees of Duke University, the University of North Dakota, and/or the University of Colorado (most recently, these included Duke University IACUC #A143‐12‐05, approved 05/25/2012, and University of North Dakota IACUC #0802‐2, approved 04/03/08). Sample collection in Madagascar was also approved by Madagascar National Parks and CITES (05US040035/9).

### 2.3 DNA extraction

We extracted DNA from the samples following the manufacturers’ instructions, using either DNA miniprep kits (Sigma, St. Louis, MO) or DNeasy^®^ Blood and Tissue kits (Qiagen, Valencia, CA). We preserved whole blood from the wild subjects on Whatman FTA^®^ Classic cards (GE Healthcare Life Sciences, Buckinghamshire, UK). From these cards, we extracted DNA and increased its quality and quantity by whole genome amplification (Repli‐G Single Cell Kit^®^, Qiagen, Valencia, CA).

### 2.4 Genotyping

To genotype individuals at the MHC‐DRB loci, we used parallel‐tagged sequencing to pool amplicons of 50–125 individuals, including two to five replicates per individual, for a total of 845 amplicons in eight PCR pools. We sequenced a 171‐bp fragment of the MHC‐DRB second exon, containing the antigen‐binding site, using three Ion Torrent PGM^®^ 314v2 chips (Life Technologies, Grand Island, NY) and five 454 Titanium^®^ 1/8th lanes (Roche, Nutley, NJ). To distinguish alleles from artifacts, we used a published workflow that relies upon relative frequency of variants within an amplicon and comparisons between replicate PCRs for each sample to identify alleles (for details, see Grogan et al., 2016). Once animals were genotyped, we determined MHC supertype by identifying nucleotide sites under positive selection or positively selected sites (PSS), via the CODEML analysis in PAML Version 4.7 (Yang, 2007). We used the physiochemical properties of PSS amino acids to determine MHC‐DRB supertypes of alleles or groups of alleles with similar binding properties at antigen‐binding sites (ABS). We used heat mapping to determine supertypes in Genesis Version 1.7.6 (See Table S1 for details regarding MHC supertypes and corresponding MHC alleles: Doytchinova & Flower, 2005; Huchard et al., 2008; Schwensow et al., 2007; Sepil et al., 2013).

### 2.5 Statistical analyses

Using an ANOVA, we compared the average MHC‐DRB diversity of captive vs. wild individuals. We investigated whether sampling effort affected the number of alleles detected in each population compared to the actual allelic richness present in each population. To do so, we conducted permutation tests by randomly sampling ten individuals per population and counting the number of unique alleles represented in this random sample. After 100 iterations, we calculated the mean and *SD* of a sampling effort of ten. We then repeated this procedure in an incremental process, adding ten to our sampling effort in each step, up to a sample of 100 individuals. We conducted these permutation tests for both the wild population (*n* = 180) and the captive DLC population (*n* = 101), and plotted the average number of MHC‐DRB alleles detected per given sampling effort. We performed all analyses in RStudio (Version 3.0.2).

To evaluate the use of nH_o_ as a proxy for MHC diversity, we used a linear regression to compare MHC‐DRB diversity to microsatellite data separately for each population, using data from previously published work (for details of microsatellite genotyping of the captive population, see Charpentier et al., 2008; for details of microsatellite genotyping of the wild population, see Pastorini et al., 2015). Briefly, 73 ring‐tailed lemurs from the DLC population were each genotyped at 10–15 microsatellite loci. From BMSR, 130 individuals captured from 2003 to 2005 were genotyped at 10 microsatellite loci. Only microsatellite loci that conformed to Hardy–Weinberg expectations were used to calculate observed nH_o_ per individual. Within the 73 captive individuals, nH_o_ ranged from 0.21 to 0.86 (mean ± *SE* = 0.56 ± 0.02; Charpentier et al., 2008). In contrast, nH_o_ for the wild population ranged from 0.33 to 1.0 (mean ± *SE* = 0.76 ± 0.14; Pastorini et al., 2015).

Lastly, to assess the influence of approximately 50 years of captivity and captive management on MHC‐DRB diversity in the DLC population, we divided our study period into decades and compared the average MHC‐DRB diversity of the infants born during each decade from 1980 to 2013 (see Charpentier et al., 2008). To disentangle the relative influences of the founder effect from genetic drift over generations, we used the microsatellite data and the linkage disequilibrium method (Laurie‐Ahlberg & Weir, 1979; Hill, 1981; Waples & Do, 2010) in NeEstimator V2 (Do et al., 2014) to estimate the effective population size for the DLC population (*n* = 80) per decade in captivity from 1980 to 2007, as well as for the wild population (*n* = 130) from 2003 to 2005. These data are somewhat limited, however, as they do not include every individual residing at the DLC or BMSR during these years, nor every individual that was genotyped for MHC‐DRB. Additionally, we lack microsatellite data from individuals born at or transferred to the DLC after 2007, as well as for all individuals residing at the Cincinnati and Indianapolis Zoos and born at BMSR after 2006.

## 3 RESULTS

**Figure 2:**
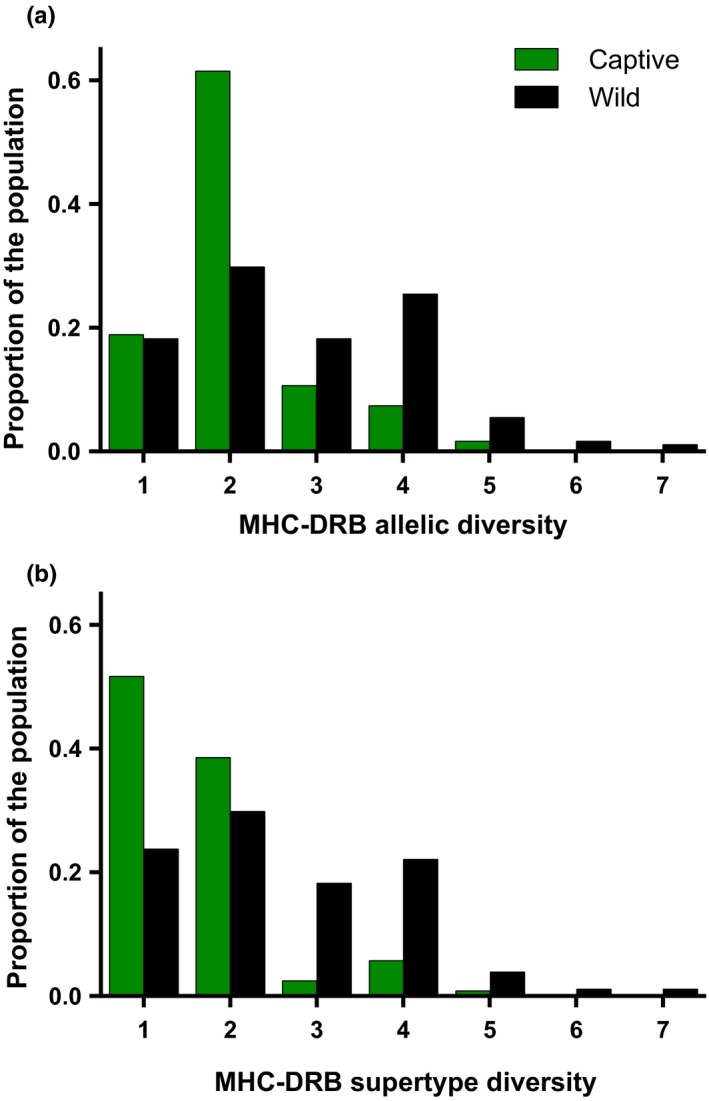
Genetic diversity in ring‐tailed lemurs showing the number of (a) MHC‐DRB alleles and (b) MHC‐DRB supertypes at the individual level for both captive (green) and wild (black) populations

In testing for differences in MHC‐DRB diversity between wild and captive populations of ring‐tailed lemurs, we found that wild individuals were significantly more diverse than were captive individuals (Figure 2). Specifically, there were significantly more MHC‐DRB alleles (*n* = 301, *F* = 23.97, *p* < .001; Figure 2a; Table 1), as well as significantly more MHC‐DRB supertypes (*n* = 301, *F* = 7.035, *p* < .001; Figure 2b; Table 1) in wild individuals compared to captive individuals. Wild individuals possessed a mean (±*SD*) of 2.78 (±1.34) alleles (range = 1–7) and 2.60 (±1.32) supertypes (range = 1–7), whereas captive individuals possessed 2.1 (±0.86) alleles (range = 1–5) and 1.65 (±0.83) supertypes (range = 1–5).

**Table 1:** Individual and population level of MHC‐DRB diversity across captive animals at three institutions compared to animals from one wild population

	Captive: Cincinnati zoo	Captive: Indianapolis zoo	Captive: DLC	Total captive population	Wild population
Number of individuals genotyped	2	18	101	121	180
Average number of alleles per individual	3.5	2.56	2.0	2.10	2.78
Total number of alleles in population	4	12	16	20	53
Number of private alleles	0	2	1	2	35
Average number of supertypes per individual	3.5	2.11	1.51	1.65	2.6
Total number of supertypes in population	4	9	11	13	24
Number of private supertypes	0	1	1	1	12

**Figure 3:**
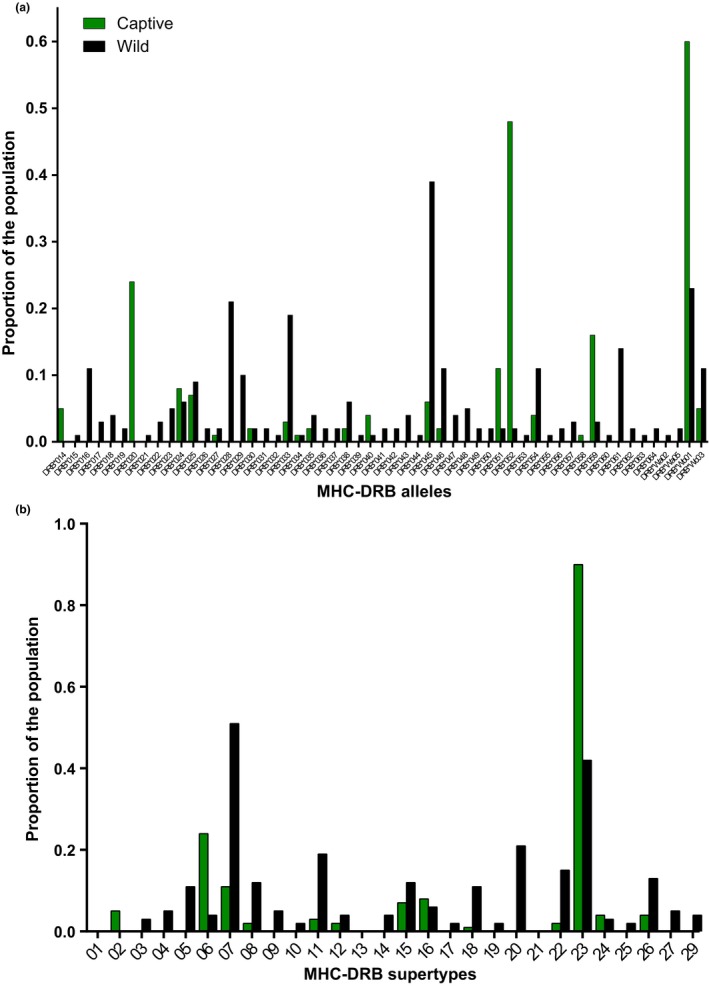
The frequency distribution of (a) MHC‐DRB alleles (Leca‐DRB) and (b) MHC‐DRB supertypes in captive (green) and wild (black) populations of ring‐tailed lemurs

The wild population also showed more allelic richness, with 52 unique MHC‐DRB alleles and 24 MHC supertypes, compared to 13 supertypes comprised of 20 MHC‐DRB alleles in the captive population (Figure 3). The two populations shared 18 alleles (12 supertypes); however, the captive population had two private alleles not found in the wild population and one private MHC supertype. In contrast, the wild population had 35 private alleles and 12 MHC supertypes that were not shared by the captive population (for details of which alleles were shared or were private, see Table S1). Neither sex nor age influenced the distribution of MHC‐DRB alleles or supertypes between populations or individuals.

Within each population, MHC‐DRB alleles and supertypes were unevenly distributed (Figure 3). Within the captive population, three alleles were present in a majority of the individuals, whereas the remaining 17 alleles were present in only a few individuals (overall range = 0.0–60.3%, overall mean = 10.6%; Figure 3a). The most abundant allele was present in more than 60% of captive individuals, and more strikingly, the most common MHC supertype was present in >90% of captive individuals (range = 1.0%–90.0%, mean = 6.0%). By contrast, the most abundant MHC‐DRB allele within the wild population was present in only 38.8% of individuals (range = 0.0–38.8%, mean = 5.2%; Figure 3b), and the most abundant supertype was present in only 51% of individuals (range = 2.0%–51.0%, mean = 9%). Thus, we found that MHC‐DRB variation in the wild population was more evenly spread than was MHC‐DRB variation in the captive population.

**Figure 4:**
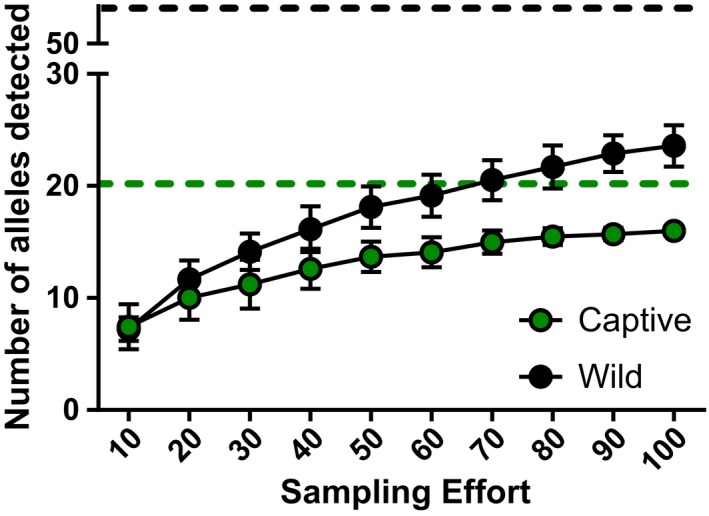
The mean number (±*SE*) of unique MHC‐DRB alleles detected in captive (green circles) and wild (black circles) populations of ring‐tailed lemurs. The unique alleles detected were dependent upon sampling effort, estimated using resampling techniques, and are compared to the number of unique alleles present in the captive (dashed green line) and wild (dashed black line) populations

Next, we calculated the average allelic richness detected for a given sampling effort (Figure 4). In the captive population, the number of unique alleles detected reached a plateau near a sampling effort of 60 individuals or 50% of the captive population sampled. When 60 captive animals were sampled, approximately 75% of 20 possible unique alleles were detected. In the wild population, however, sampling 55% of the population detected only 44% (*n* = 23) of the 52 unique MHC‐DRB alleles present. Therefore, the more diverse a population, the greater the sampling effort required to detect the majority of the MHC diversity present.

**Figure 5:**
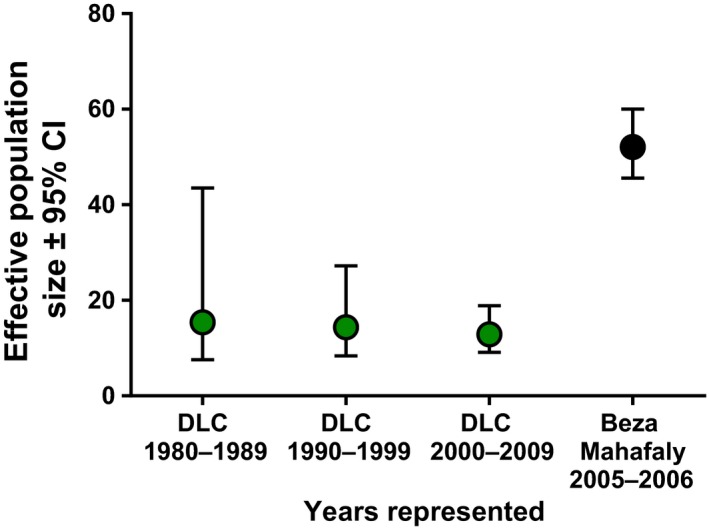
Effective population size (*N*_e_) at the DLC and BMSR, calculated using the linkage disequilibrium method. Genotyped infants from the DLC (in green circles) were grouped according to individuals born within each decade from 1980 through 2010, whereas *N*_e_ from BMSR (represented by a black circle) was estimated from animals residing in the reserve between 2005 and 2006. Upper and lower limits indicate 95% confidence intervals calculated using the jackknife method

**Figure 6:**
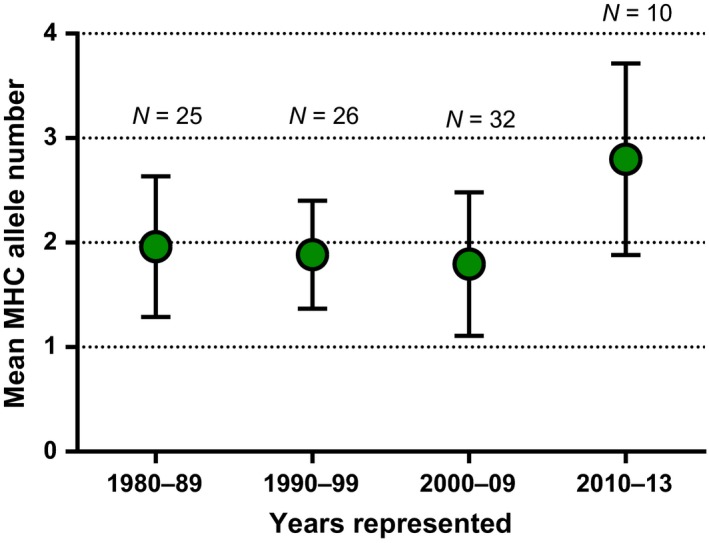
Mean MHC‐DRB allelic richness (±*SD*) possessed by ring‐tailed lemur infants born at the DLC, separated typically by decade of birth from 1980 through the birth season of 2013. The number of infants born per period is shown above each data point

In our comparison between neutral and functional diversity, we found nH_o_ to be significantly and positively correlated with the number of MHC‐DRB alleles (*n* = 71, *F* = 5.884, *p* = .018) in the captive population; however, variation in nH_o_ explained little of the variation in the number of MHC‐DRB alleles (adjusted R^2^ = 0.065, slope = 1.26). Conversely, in the more diverse wild population, MHC diversity was not correlated with nH_o_ (*n* = 88, *F* = 0.002, *p* = .962). Consequently, although average nH_o_ decreased significantly across the decades from 1980 to 2010 (see Charpentier et al., 2008), MHC‐DRB diversity in infants born at the DLC remained constant (Spearman correlation, *n* = 83, r = −0.11, *p* = .28). Despite careful management, the estimated effective population sizes decreased from 15.4 individuals during 1980–1989 to 14.4 individuals during 1990–1999, then down to 12.9 individuals during 2000–2009. In contrast, the effective population size from 2003 to 2005 at BMSR was 52.1 individuals (Figure 5). After 2010, however, MHC‐DRB diversity in captive‐born infants increased significantly (Spearman correlation, *n* = 93, r = 0.35, *p* < .001; Figure 6) compared to the MHC‐DRB diversity of infants born in the decades prior to 2010. In fact, the MHC‐DRB diversity of infants born after 2010 (mean ± *SD* = 2.8 ± 0.92) was comparable to the average MHC‐DRB diversity of individuals in the wild (*F* = 0.002, *p* = .966).

## 4 DISCUSSION

In a comparison of two populations of ring‐tailed lemurs, both individual‐ and population‐level MHC‐DRB diversity were greater in wild animals than in captive animals. Individuals from among the wild lemurs possessed, on average, one allele more than did individuals from among the captive lemurs. The number of unique MHC‐DRB alleles and MHC‐DRB supertypes present in the wild population was more than twice the number in the captive population at the three institutions included in this study. In both the wild and captive populations, allelic frequency showed a skewed distribution, as over 50% of MHC‐DRB alleles present in each population were found in five or fewer individuals. Just as some microsatellite variation already has been lost in captivity (Charpentier et al., 2008), these rare MHC‐DRB alleles are at greater risk of being lost via genetic drift than they would be in a larger population. In the captive population, the decreased genetic diversity likely reflects effects of 1) a genetic bottleneck, owing to only a few founding individuals having been initially captured from the wild, 2) decades of reduced gene flow between institutions, and 3) genetic drift, especially in small populations. Without the subsequent introduction of new MHC alleles, captive management efforts at the DLC, based on pedigree data, did not counteract the loss of genetic diversity over four decades (Charpentier et al., 2008); however, we found evidence of a genetic rescue occurring via the transfer of new individuals to the DLC after 2010.

Genetic drift may, in fact, have a stronger effect on immune system or functional genes than on neutral genetic diversity (Bateson, Whittingham, Johnson, & Dunn, 2015; Froeschke & Sommer, 2014; Marsden et al., 2012) or may overwhelm the influence of selection in small populations (Luo, Pan, Liu, & Li, 2012; Miller & Lambert, 2004). In some cases, MHC diversity even declines faster than does neutral diversity (Bateson et al., 2015; Bollmer et al., 2011; Ejsmond & Radwan, 2009; Eimes et al., 2011; Sutton et al., 2011, 2015); however, we saw a more rapid decline of neutral diversity compared to MHC diversity in the captive ring‐tailed lemur population at the DLC. Thus, although genetic drift has resulted in decreased neutral diversity in the DLC population, selective pressure may be mitigating the loss of immunogenetic diversity in captivity (Ellison et al., 2012; Newhouse & Balakrishnan, 2015). Captivity may even be exerting strong selective pressure through exposure to novel pathogens not previously experienced during millions of years of isolation on Madagascar.

Although limited MHC diversity does not necessarily condemn a species to extinction (e.g., Castro‐Prieto, Wachter, & Sommer, 2011; Lau, Jaratlerdsiri, Griffith, Gongora, & Higgins, 2014; Plasil et al., 2016; Weber, Stewart, Schienman, & Lehman, 2004; Weber et al., 2013; Zhu, Ruan, Ge, Wan, & Fang, 2007), low MHC diversity can compromise population viability over the long term by increasing susceptibility to disease (Belov, 2011; Radwan, Biedrzycka, & Babik, 2010; Segelbacher et al., 2014; Spielman, Brook, Briscoe, & Frankham, 2004). In particular, many species are likely to increasingly encounter novel pathogens in the future because of increased exposure to humans, their livestock, other domesticated animals (e.g., feral dogs and cats), and invasive species (Barrett, Brown, Junge, & Yoder, 2013; Daszak, Cunningham, & Hyatt, 2000, 2001). The negative effects of low MHC diversity on an animal's ability to combat novel pathogens also may be exacerbated by stress from human disturbance or environmental perturbations (Frankham, 2005b). These perturbations to the environment, which often decrease population size (e.g., a drought: Gould et al., 2003), are likely to subject populations to increasing effects of genetic drift, which may further decrease genetic diversity in a negative feedback loop that subsequently renders a population even more susceptible to novel pathogens. Thus, quantifying the current level of MHC diversity, especially in wild or endangered species, is essential to future conservation efforts.

Although previous researchers have estimated genetic diversity using nH_o_ as a proxy for functional diversity (Höglund, Wengström, Rogell, & Meyer‐Lucht, 2015; Segelbacher et al., 2014), we found only a weak correlation between MHC‐DRB diversity and nH_o_ in captivity and no correlation in our wild population. Our MHC and nH_o_ findings, however, confirm that captive individuals have less overall genetic diversity than do wild individuals (Charpentier et al., 2008; Pastorini et al., 2015). Captive ring‐tailed lemurs are more closely related within institutions than between institutions, and also have greater relatedness between individuals at a single institution than do individuals within a social group at the BMSR (Pastorini et al., 2015). Together, the MHC and nH_o_ data indicate that groups of ring‐tailed lemurs at some institutions have historically functioned as independent, small populations, exhibiting less gene flow than typically occurs between wild groups (Pastorini et al., 2015). In other words, there are fewer transfers between captive groups at the institutions examined in this study than there are immigrations between wild groups. Between 1980 and 2013, the AZA SSP included 2,839 ring‐tailed lemurs housed at 289 institutions. During this period, 1,168 transfers, including some animals that were transferred multiple times, occurred between 217 institutions, which is a rate of 0.1 transfers per institution per year (Gina Ferrie, personal communication). In contrast, researchers at the BMSR and Berenty Reserve have observed an average of 1.4 and 2.9 transfers, respectively, per social group annually (Koyama, Nakamichi, Ichino, & Takahata, 2002; Sauther, 1991; Sussman, 1992).

In captivity, these transfers occur for several reasons, most importantly as part of the AZA SSP management of diversity of the captive population in the USA. The ring‐tailed lemur population including all individuals from all AZA SSP institutions is a large and successfully managed population, considered a Green SSP Program because the population has retained “a minimum of 90% gene diversity at 100 years or 10 generations, and includes at least 50 animals held among at least three AZA member institutions” (AZA 2014). Estimates of genetic diversity are based on the number of founder lineages represented in the population, rather than on molecular estimates via microsatellites or allelic diversity of SNPs or candidate genes (AZA Population Management Center Population Management Guidelines; Lacy, 1995). Unfortunately, little is known about the origins or relationships of the founding ring‐tailed lemurs in the captive population, and molecular work has not confirmed that founders were unrelated to one another. AZA SSP management has prevented some loss of diversity through genetic drift; however, these efforts have not been completely successful at maintaining levels of neutral or adaptive diversity equivalent to the level of genetic diversity seen in the wild. Given our findings regarding the limited correlation between neutral and adaptive diversity, we recommend the inclusion of molecular data, particularly of potentially important candidate genes, into the Breeding and Transfer Plan. We also recommend increased transfer of key individuals possessing unique MHC‐DRB alleles or substantial MHC‐DRB diversity, particularly between international institutions that may hold unique individuals from different source populations (Frankham, 2015; Ivy, 2016; Pastorini et al., 2015). The inclusion of molecular data and the movement of endangered species across international borders may be financially and logistically constrained, but could increase the diversity of captive offspring and preserve extremely rare MHC‐DRB alleles within the captive population.

Additionally, although uneven MHC‐DRB allelic frequencies are present in other vertebrate species (Alcaide, Muñoz, Martínez‐de la Puente, Soriguer, & Figuerola, 2014; Pechouskova et al., 2015; Weber et al., 2013), the highly skewed distribution of MHC‐DRB alleles in ring‐tailed lemurs increases the likelihood that rare, potentially important alleles may have been lost, particularly from the captive population, due to genetic drift. The diversity and distribution of MHC‐DRB in animals across institutions should be evaluated and that assessment incorporated into breeding recommendations. To gain an accurate picture of MHC‐DRB diversity in populations, especially captive ones, sampling should include almost every individual because of the abundance of rare alleles and uneven distribution.

Lastly, we show that captive populations suffer from the effects of small founder population and possible genetic drift, but that careful management, including the influx of new individuals, can negate this potential threat and lead to an increase in the number of MHC‐DRB alleles. This kind of genetic “rescue” by a few individuals is one method of increasing genetic diversity, viability, and survivorship (e.g., Adams, Vucetich, Hedrick, Peterson, & Vucetich, 2011; Johnson et al., 2010; Westemeier et al., 1998; reviewed in Frankham, 2015). Without continued introduction of new alleles, however, the increase in individual diversity may be temporary (Hedrick, Peterson, Vucetich, Adams, & Vucetich, 2014). The inclusion of measures of genetic diversity into conservation management plans is a critical step in the preservation of viable populations, both in captivity and in the wild (Witzenberger & Hochkirch, 2011).

MHC‐DRB is not the only component of immunogenetic diversity that could be monitored and managed, however. For example, toll‐like receptors could provide an informative comparison to the MHC‐DRB. Whereas the MHC controls the activation of the adaptive immune system, toll‐like receptors are essential to the innate immune response through intracellular signaling (Gonzalez‐Quevedo, Phillips, Spurgin, & Richardson, 2015; Grueber et al., 2015; Morris, Wright, Grueber, Hogg, & Belov, 2015). Alternatively, assessment of functional immmunogenetic diversity could be expanded using next‐generation Ig‐seq technology to explore expressed antibodies (Larsen, Campbell, & Yoder, 2014). This method allows for monitoring of disease status of wild populations and surveillance of novel diseases, an important component of advanced conservation management.

As an endangered strepsirrhine endemic to Madagascar, the ring‐tailed lemur is both a flagship conservation species for one of the world's top biodiversity hot spots and a prime example of a species in peril (Gould & Sauther, 2016; LaFleur et al. 2016). Like all nonhuman primates, ring‐tailed lemurs face significant anthropogenic threats (Reuter et al. 2017; Schwitzer et al., 2013) and are increasingly susceptible to environmental change through loss of genetic diversity (Frankham, 2005a, 2005b; Charpentier et al., 2008; Spielman et al., 2004). Studies such as this one are key for assessing a species’ ability to respond to these threats, including novel pathogens, as well as to adapt to changing climatic conditions. Such studies are also crucial for determining whether captive populations are sufficiently representative of wild populations to serve as a reservoir for the purpose of reintroduction or repopulation.

## CONFLICT OF INTEREST

The authors declare no conflict of interests.

## AUTHOR CONTRIBUTIONS

CMD and KEG conceived the study and participated in study design and sample collection. KEG performed the molecular work and data analyses, as well as drafted the manuscript. CMD critically revised the manuscript drafts. MLS and FPC coordinated and collected the wild samples from Madagascar, as well as edited the manuscript. All authors approved the final manuscript.

## DATA ACCESSIBILITY

The genotypes of each ring‐tailed lemur, as well as location of origin, have been deposited in Dryad: https://doi.org/10.5061/dryad.bn7h0.
