# Supplementary material for: Genetic wealth, population health: Major histocompatibility complex variation in captive and wild ring‐tailed lemurs (Lemur catta)
Source: Ecol Evol. 2017 Aug 17;7(19):7638–49. doi: 10.1002/ece3.3317 (PMC5632616; doi:10.1002/ece3.3317)
Supplement: Supplementary file 1 [file ECE3-7-7638-s001.docx]

**Appendix Table. MHC supertypes and the corresponding MHC alleles.**

| **MHC Supertype** | **Supertype Population Presence** | **MHC Allele** | **Allele Population Presence** | **Accession Number** |
| --- | --- | --- | --- | --- |
| **Supertype 01** | Neither | Leca-DRB*Wa01 | Neither | AB078265 |
| **Supertype 02** | Captive only | Leca-DRB*014 | Captive only | KJ817213 |
| **Supertype 03** | Wild only | Leca-DRB*017 | Wild only | KJ817216 |
| **Supertype 04** | Wild only | Leca-DRB*021  Leca-DRB*023 | Wild only  Wild only | KJ817220  KJ817222 |
| **Supertype 05** | Wild only | Leca-DRB*016 | Wild only | KJ817215 |
| **Supertype 06** | Captive & Wild | Leca-DRB*020  Leca-DRB*022  Leca-DRB*Wa02 | Captive only  Wild only  Wild only | KJ817219  KJ817221  AB078287 |
| **Supertype 07** | Captive & Wild | Leca-DRB*042  Leca-DRB*045  Leca-DRB*Wc03 | Wild only  Captive & Wild  Captive & Wild | KJ817241  KJ817244  AB078279 |
| **Supertype 08** | Captive & Wild | Leca-DRB*038  Leca-DRB*039  Leca-DRB*050  Leca-DRB*057 | Captive & Wild  Wild only  Wild only  Wild only | KJ817237  KJ817238  KJ817249  KJ817256 |
| **Supertype 09** | Wild only | Leca-DRB*043  Leca-DRB*044 | Wild only  Wild only | KJ817242  KJ817243 |
| **Supertype 10** | Wild only | Leca-DRB*037 | Wild only | KJ817236 |
| **Supertype 11** | Captive & Wild | Leca-DRB*033  Leca-DRB*034  Leca-DRB*036 | Captive & Wild  Captive & Wild  Wild only | KJ817232  KJ817233  KJ817235 |
| **Supertype 12** | Captive & Wild | Leca-DRB*032  Leca-DRB*035 | Wild only  Captive & Wild | KJ817231  KJ817234 |
| **Supertype 13** | Neither | Leca-DRB*Wb02 | Neither | AB078229 |
| **Supertype 14** | Wild only | Leca-DRB*018 | Wild only | KJ817217 |
| **Supertype 15** | Captive & Wild | Leca-DRB*015  Leca-DRB*025  Leca-DRB*026 | Wild only  Captive & Wild  Wild only | KJ817214  KJ817224  KJ817225 |
| **Supertype 16** | Captive & Wild | Leca-DRB*024 | Captive & Wild | KJ817223 |
| **Supertype 17** | Wild only | Leca-DRB*Wa05 | Wild only | AB078292 |
| **Supertype 18** | Captive & Wild | Leca-DRB*027  Leca-DRB*029 | Captive & Wild  Wild only | KJ817226  KJ817228 |
| **Supertype 19** | Wild only | Leca-DRB*019 | Wild only | KJ817218 |
| **Supertype 20** | Wild only | Leca-DRB*028 | Wild only | KJ817227 |
| **Supertype 21** | Neither | Leca-DRB*Wb04 | Neither | AB078248 |
| **Supertype 22** | Captive & Wild | Leca-DRB*046  Leca-DRB*047 | Captive & Wild  Wild only | KJ817245  KJ817246 |

| **MHC Supertype** | **Supertype Population Presence** | **MHC Allele** | **Allele Population Presence** | **Accession Number** |
| --- | --- | --- | --- | --- |
| **Supertype 23** | Captive & Wild | Leca-DRB*031  Leca-DRB*051  Leca-DRB*052  Leca-DRB*053  Leca-DRB*058  Leca-DRB*059  Leca-DRB*061  Leca-DRB*Wb01 | Wild only  Captive & Wild  Captive & Wild  Wild only  Captive only  Captive & Wild  Wild only  Captive & Wild | KJ817230  KJ817250  KJ817251  KJ817252  KJ817257  KJ817258  KJ817260  AB078199 |
| **Supertype 24** | Captive & Wild | Leca-DRB*030  Leca-DRB*040  Leca-DRB*060 | Captive & Wild  Captive & Wild  Wild only | KJ817229  KJ817239  KJ817259 |
| **Supertype 25** | Wild only | Leca-DRB*049 | Wild only | KJ817248 |
| **Supertype 26** | Captive & Wild | Leca-DRB*054  Leca-DRB*056  Leca-DRB*062  Leca-DRB*064 | Captive & Wild  Wild only  Wild only  Wild only | KJ817253  KJ817255  KJ817261  KJ817263 |
| **Supertype 27** | Wild only | Leca-DRB*048 | Wild only | KJ817247 |
| **Supertype 28** | Wild only | Leca-DRB*041  Leca-DRB*055  Leca-DRB*063 | Wild only  Wild only  Wild only | KJ817240  KJ817254  KJ817262 |
